# Characterization of gelatin/chitosan ploymer films integrated with docosahexaenoic acids fabricated by different methods

**DOI:** 10.1038/s41598-019-44807-x

**Published:** 2019-06-10

**Authors:** Luyun Cai, Hang Shi, Ailing Cao, Jingze Jia

**Affiliations:** 1 College of Life Sciences, China Jiliang University, Hangzhou, Zhejiang 310018 China; 2 National & Local Joint Engineering Research Center of Storage, Processing and Safety Control Technology for Fresh Agricultural and Aquatic Products, Bohai University, Jinzhou, 121013 China; 3 Hangzhou Customs District, Hangzhou, 310007 China

**Keywords:** Proteins, Health care

## Abstract

In this study, docosahexaenoic acid powder-enhanced gelatin-chitosan edible films were prepared by casting, electrospinning and coaxial electrospinning, respectively. The color (CR), transparency (UV), light transmission (UV), mechanical strength (TA-XT), thermal stability (DSC), crystalline structures (XRD), molecular interactions (FTIR), and microstructure (SEM) were assessed in the analytical research. The results of the research showed that the electrospinning process and the coaxial electrospinning process produced a smooth surface visible to by the naked eye and a uniform granular network structure in a unique film-forming manner, thereby exhibiting good water solubility and mechanical properties. In contrast, the casted film was smooth, transparent, and mechanically strong but poorly water soluble. It was also found that the addition of docosahexaenoic acid powder affected the optical, physical and mechanical properties of the film to varying degrees.

## Introduction

Electrospinning is a simple and reliable technique for preparing nanofibers and can be used for a variety of polymer materials. The resulting nanofibers have a smooth surface and controlled morphology^1^. Coaxial electrospinning can produce micro/nano fibers with a core-shell structure and highly different compositions, which is an improvement over simple electrospinning^2^. The nanofiber particles created by electrospinning and coaxial electrospinning are ideal for many applications such as biosensors, enzyme immobilization, drug delivery, scaffold tissue engineering, wound healing, and so on^3,4^. The difference is that casting involves the application of a polymer solution on a substrate and then removal of the solvent to cause molecular orientation of the polymer molecules, resulting in film formation^5^. However, studies on the characterization of nanoparticles polymer films fabricated by different methods, especially coaxial electrospinning have been scarce. As a biopolymer, we selected gelatin obtained from flounder, which has been FDA approved. *Paralichthys orbignyanus*, also known as the flounder, is an economically important fish that lives in temperate water and rich in polyunsaturated fatty acids. Current research on flounders has mainly focused on farming, breeding and rough machining^6^. A large amount of waste materials, such as fish skin and bones, are left during the processing of flounder, especially the bones, which account for about 40% of the whole fish. Therefore, flounder bones were used as the material from which to extract and purify gelatin by the hot-water extraction method.

Edible films used in drug and food packaging are biodegradable, naturally renewable and edible biopolymers. Polysaccharides and proteins are used in various fields as a basic excellent macromolecule substance. The typical combination gelatin-chitosan (GC) biocomposite membrane is a type of “green packaging” material with excellent development potential that has been studied by some researchers^7–9^. Gelatin molecules are rich in biological functional groups such as carboxyl, hydroxyl, and amino groups that have excellent biocompatibility and are potential film-forming materials. Chitosan is the only natural polysaccharide with a large amount of basic aminopolysaccharide, which is non-toxic, degradable, bacteriostatic, and biocompatible and has good film forming properties^10^. In recent years, to change the performance and characterization of GC films, functional ingredients such as inorganic salts, phenolic compounds, rosmarinic acid and ginger essential oils have been studied by some researchers^11–14^. However, docosahexaenoic acid (DHA) combined with polysaccharides-proteins has not been studied in recent reports. DHA is a major substance in the growth and maintenance of nervous system cells and an important component of the brain and retina^15^. Supplementing ω-3 polyunsaturated fatty acids can effectively reduce some risk of disease in the long term. The scientific novelty of the work is that we found that fish oil full of ω-3 polyunsaturated fatty acids combined with protein to form the emulsions but seldom films^16^. Therefore, we selected DHA algal oil powder as the core material and functional ingredient, combined with coaxial electrospinning and other processes to prepare membrane materials with good biocompatibility and safety and then to compare their structure and physicochemical properties; we hope the result can be used in the food packaging and biomedical fields.

The essential objective of the present research was to compare the properties of edible films formed by different methods for better delivery of ω-3 polyunsaturated fatty acids and analyze the influence and interaction of macromolecular nutrient complexes on edible film. In this study, we used geltain-chitosan as the wall material and mixed or embedded docosahexaenoic acids powders as the core material, using casting, electrospinning, and coaxial electrospinning to form 5 different films. The structure and physicochemical properties were analyzed and studied to provide a scientific basis for the development and utilization of a new gelatin edible film and for industrial application.

## Results

### Water solubility and color measurement of the films

**Figure 1:**
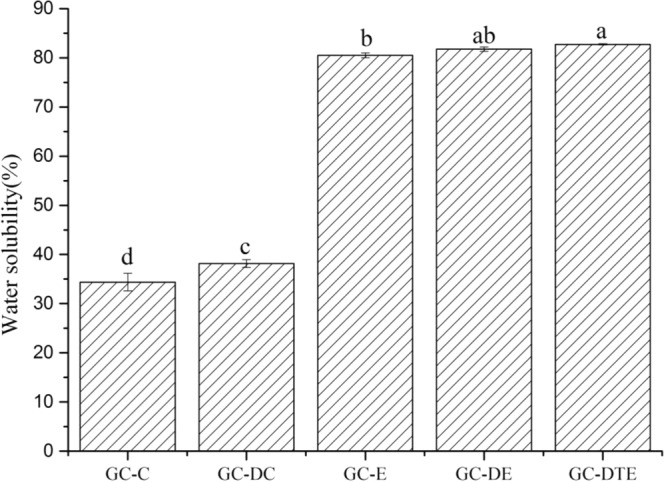
Water solubility of the five different edible films. “a–d” letters indicate significant differences (p < 0.05). Error bars show standard deviation.

Figure 1 depicts different film formation methods using a source component with a water-soluble of DHA fortified sample and a control sample. As the figure shows the films formed by electrospinning had a significantly higher water solubility than the films formed by the casting process. The slight difference between GC-E and GC-DE was the presence of DAS, which was water soluble, so GC-DC had higher water solubility than GC-C. However, with DAS as the core material wrapped with GCS, GC-DTE had a slight higher water solubility than GC-DE.

The L* and ΔE* values which were showed in Table 1 of GC-C were significantly higher than those of GC-DC, different from the films formed by electrospinning; GC-DE had the highest values, and those of GC-DTE were similar to those of GC-E and took second place. GC-C had the highest L* value, indicating better film transparency. Thus, it is believed that a DHA-enriched edible film source compound can cause increased brightness and improved visual quality.

**Table 1:** Chromatic aberation and transparency of the five different edible films.

Sample	Chromatic aberation				Transparency
	L*	a*	b*	ΔE*	
GC-C	90.12 ± 0.44^a^	−5.88 ± 0.08^c^	13.50 ± 0.35^b^	86.49 ± 0.49^a^	0.072 ± 0.01^d^
GC-DC	85.75 ± 0.98^b^	−4.71 ± 0.06^a^	16.63 ± 0.88^a^	82.54 ± 0.78^b^	0.067 ± 0.01^e^
GC-E	76.59 ± 0.74^c^	−5.27 ± 0.12^b^	7.45 ± 0.21^d^	72.52 ± 0.74^c^	1.719 ± 0.01^a^
GC-DE	88.11 ± 0.67^a^	−5.28 ± 0.07^b^	8.24 ± 0.24^d^	83.95 ± 0.56^b^	0.802 ± 0.01^c^
GC-DTE	77.56 ± 0.58^c^	−6.51 ± 0.01^d^	10.69 ± 0.11^c^	73.73 ± 0.58^c^	1.211 ± 0.02^b^

Values are mean ± standard deviation. “a–e” letters indicate significant differences (p < 0.05).

### Transparency and light transmission

**Figure 2:**
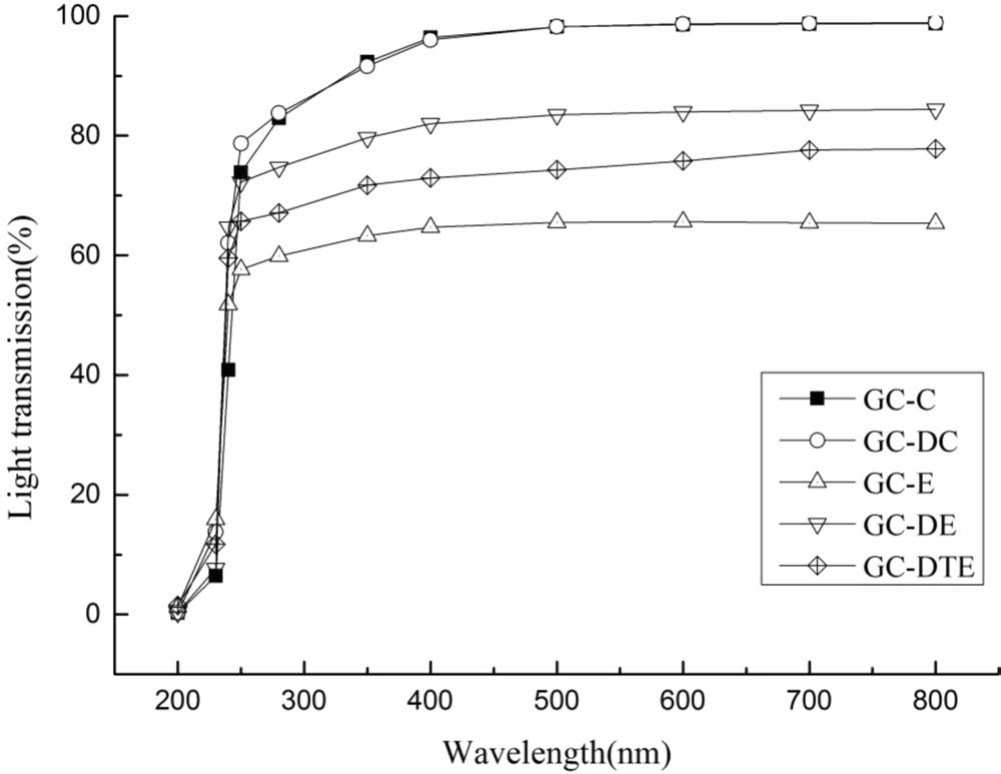
Light transmission of the five different edible films.

GC-C had similar light transmittance to that of with GC-DC, indicating that the addition of DAS had no major effect on the films made by casting, but it had a large influence on electrospinning, where the transmittance of GC-DE was higher than those of GC-DTE and GC-E. The addition of DAS in electrospinning could significantly increase the light transmittance, which may cause varying degrees of light scattering as a result of the DAS distributed throughout the film. In the visible region of 350–800 nm, the transparency of the edible films gradually stabilized with increased wavelengths of ultraviolet light. As shown in Fig. 2, the transmittance of visible light was greater than 95% and nearly 100% for casted films at 400–800 nm, whereas the transmittance was at 60–85% for electrospun films.

### Mechanical strength

**Figure 3:**
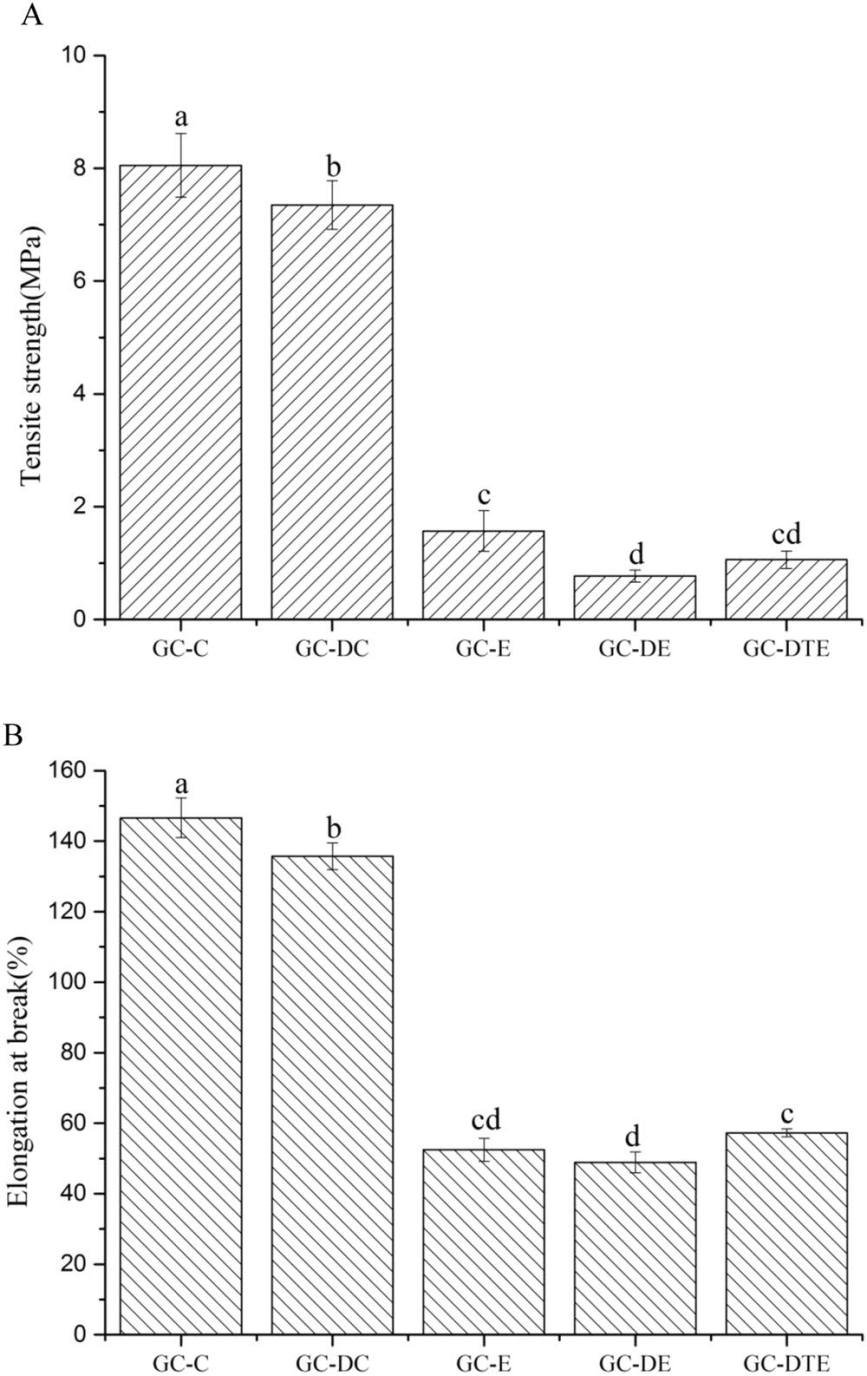
Tensite strength (**A**) and Elongation at break (**B**) of the five different edible films. “a–d” letters indicate significant differences (p < 0.05). Error bars show standard deviation.

There were large differences in the mechanical strength of films formed in different ways, and blends of DAS with different methods could be useful to influence the tensile properties. The elongations at the break and tensile strengths of edible films are shown in Fig. 3. The GC-C and GC-DC films produced a higher elongation at break of 146.6% and 135.7%, respectively, demonstrating good flexibility and deformability (Fig. 3B). Moreover, the GC-E, GC-DE and GC-DTE films had lower elongation at break than those of the casted films at 52.4%, 48.9%, and 57.2%, respectively (Fig. 3B). Thus, we concluded that the flexibility and strength of the edible films can be modified by different methods. However, we think that adding DAS to films produced different flexible films, which might endow them with wider applications.

### Different scanning calorimetry and X-ray diffraction

**Figure 4:**
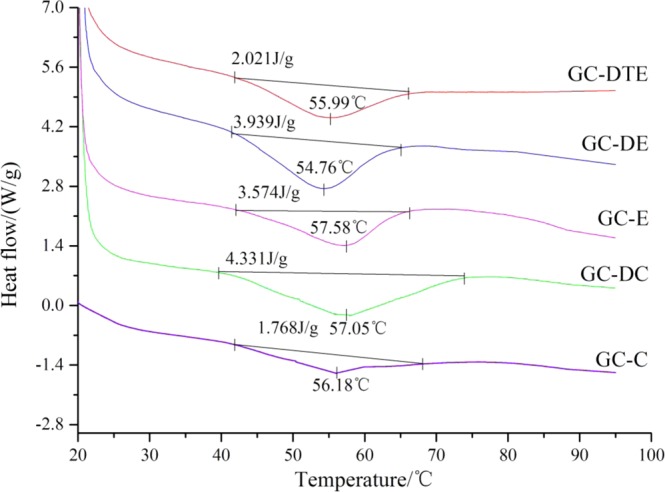
DSC curves of the five different edible films.

**Figure 5:**
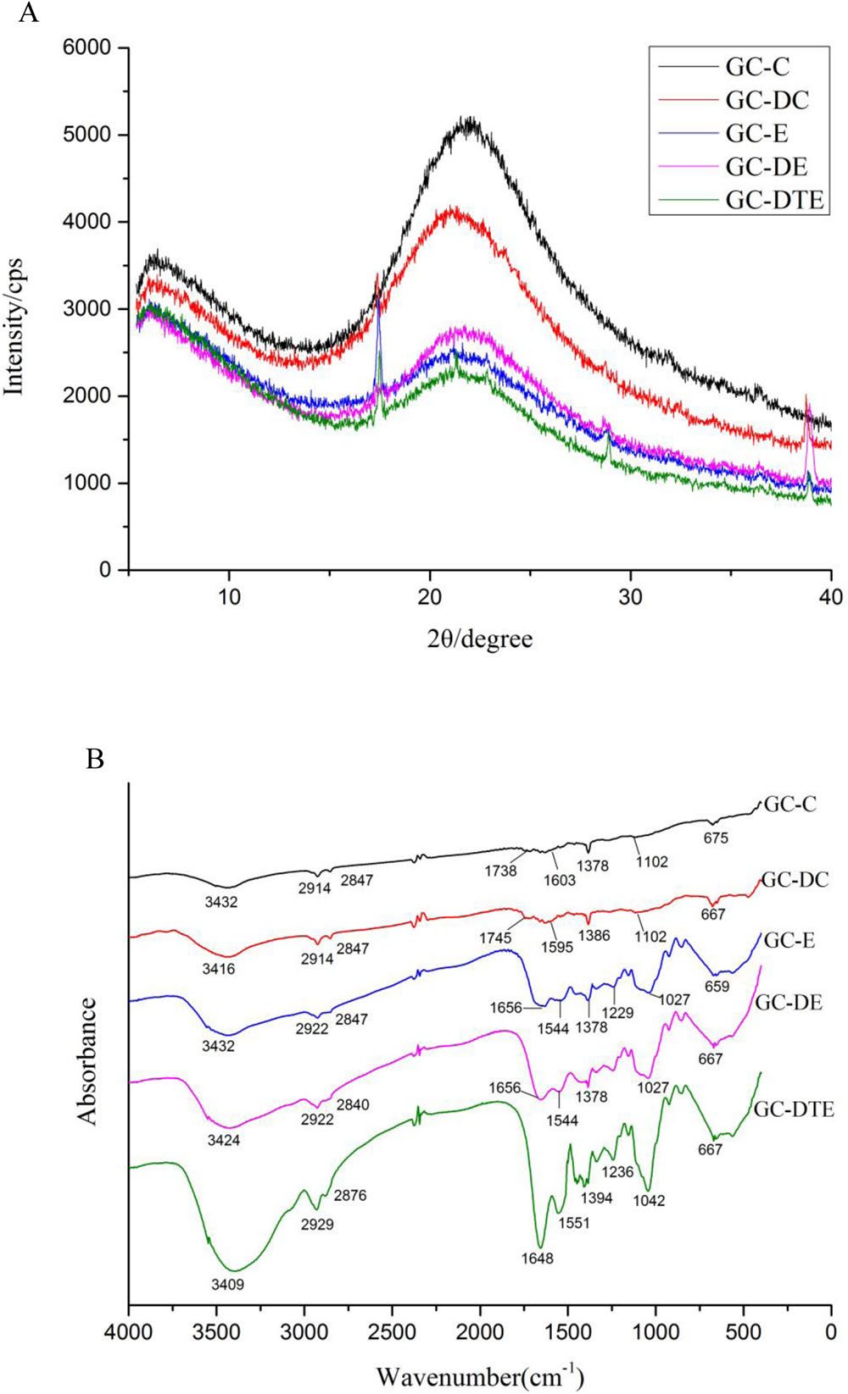
X-ray profiles (**A**) and FTIR spectra (**B**) of the five different edible films.

DSC thermal analysis of the films produced by the different methods is shown in Fig. 4. The enthalpy values of GC-C, GC-DC, GC-E, GC-DE and GC-DTE were 1.768, 4.331, 3.574, 3.939 and 2.021 J/g, and the melting temperatures of the GC-C, GC-DC, GC-E, GC-DE and GC-DTE were 56.18, 57.05, 57.58, 54.76 and 55.99 °C respectively. The crystalline structures of the edible films were characterized by X-ray diffraction (XRD) (Fig. 5A). All edible films had similar observed diffraction patterns, with a narrow peak at 6.36° and a broad peak at 21.66°, and we used the Bragg equation d(Å) = λ/2sinθ (λ = 1.54 Å) to calculate the minimum values (d) of the repeat spacings, corresponding to the interplanar spacing of 13.88 Å and 4.10 Å^17^. The research results proved that the electrospinning technology promoted the formation of amorphous structure of polymers and hindered crystallization^18^. The XRD spectra of GC-C and GC-DC showed 2 wider peaks, the strength was significantly higher, and strong intermolecular hydrogen bonds led to intramolecular binding crystallinity.

### Fourier transform infrared spectroscopy

The Fourier transform infrared spectroscopy (FTIR) spectra of the 5 different edible films are shown in Fig. 5B, and the FTIR data are shown in detail in Table 2. The 5 different edible films displayed similar main peaks, but the amplitude varied dramatically, with some of them moving. FTIR spectra are very effective in studying hydrogen bonding and compatibility, allowing observation of the FTIR spectra attributable to hydrogen bonding and the strength of hydrogen bonding. Figure 5B shows that the peak intensities of the electrospun films were better than those of the casted films, and the coaxial electrospun films were the best. The peak intensity of the films with DAS was better than that of the films without DAS. Therefore, we confirmed that the addition of DAS to and use of coaxial electrospinning with gelatin-chitosan film can facilitate uniform mixing in the film.

**Table 2:** Location and assignment of the peaks identified in FTIR spectra for the five different edible films.

Region	Peak wavenumber (cm^−1^)					Assignment and remarks
	GC-C	GC-DC	GC-E	GC-DE	GC-DTE	
Amide A	3432	3416	3432	3424	3409	N–H stretch coupled with hydrogen bond
Amide B	2914	2914	2922	2922	2929	CH antisymmetric and symmetric stretching
	2847	2847	2847	2840	2876	CH antisymmetric and symmetric stretching
Amide I	1738	1745	1656	1656	1648	C=O stretch/hydrogen bond coupled COO^−^
Amide II	1603	1595	1544	1544	1551	NH bend coupled with CN stretch
	—	—	1453	1438	1446	CH_2_ bending (scissors) vibration
	1378	1386	1378	1378	1394	CH_2_ wag of proline and glycine
Amide III	—	—	1229	1229	1236	NH bend stretch coupled C–N stretch
Fingerprint	1102	1102	1027	1027	1042	C–O skeletal stretch
	—	—	929	929	929	C-H deformation vibration (carbohydrate)
	—	—	861	854	854	C–H deformation vibration (carbohydrate)
	675	667	659	667	667	C–C Skeletal stretch

### Scanning electron microscope

**Figure 6:**
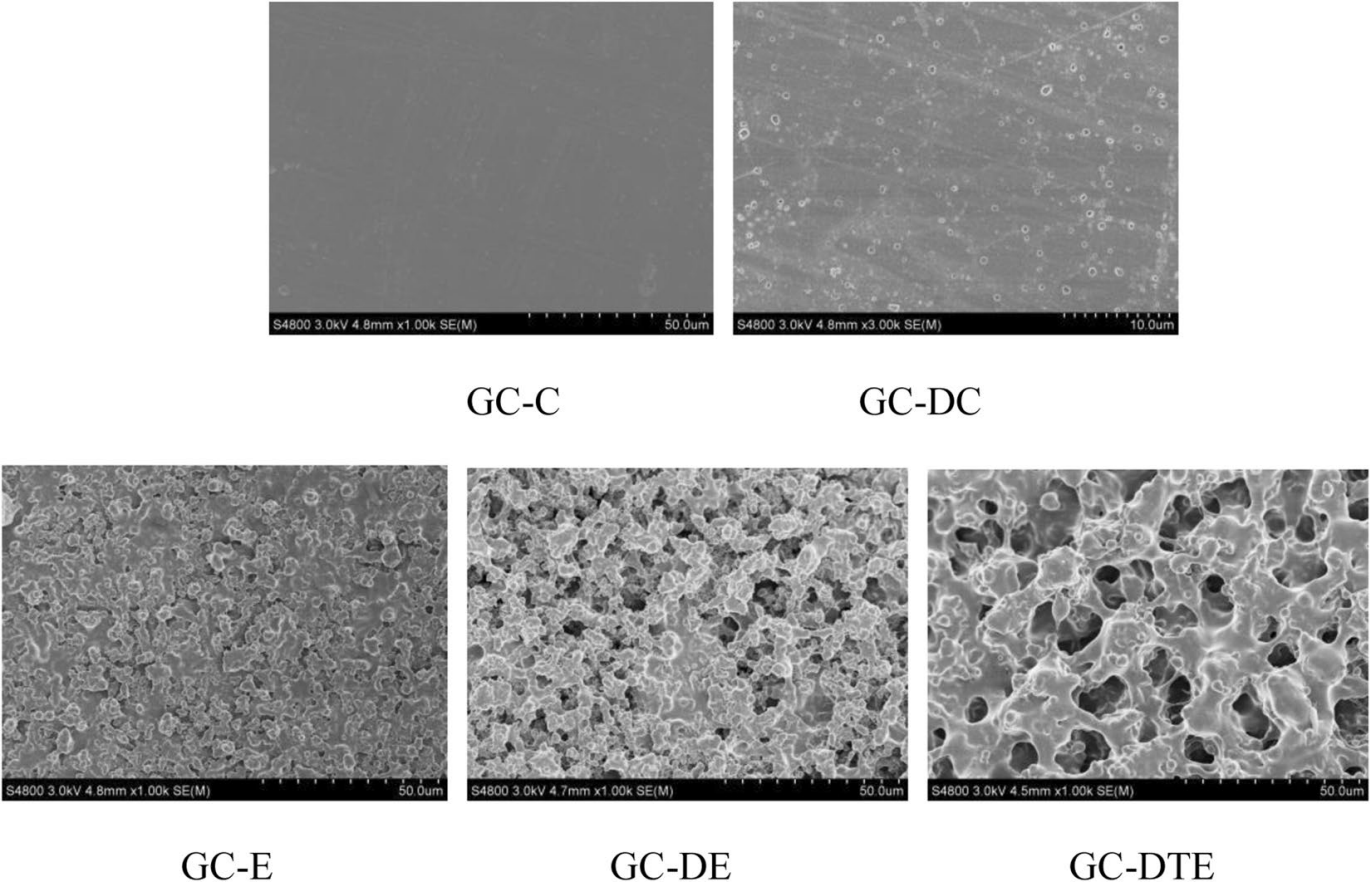
SEM images of the five different (GC-C, GC-DC, GC-E, GC-DE, GC-DTE) edible films.

Visually, the casted films were solid and semitransparent, whereas the electrospun films appeared to be white. Figure 6 shows that the surface of GC-C and GC-DC was smooth, the interface morphology was slightly rough, fine cracks were present, and no obvious wrinkles and bubbles were present, indicating that the molecules were uniformly dispersed and the film-forming effect was good. The surface of GC-E, GC-DE, and GC-DTE was uneven, composed of fiber beads, with obvious uniform pores and bulges, but the blending of materials formed a uniform dispersion system, the mesh surface was relatively tight, and the texture was relatively regular.

## Discussion

One of the most important parameters to determine the stability and quality of edible film is water solubility^19^. In terms of casted films, GC-C and GC-DC presented a smooth surface morphology and several small pores caused by solvent evaporation, indicating that the addition of chitosan could significantly improve the water resistance of the mixture membrane, and the membrane matrix polypeptide chain changed significantly. Such interference may cause a significant impediment to the gelatin molecules’ capacity to interact with water; it would be the primary responsibility of the composite film to reduce water solubility^20^. In contrast, the polymer injector and collector acceleration, because the electrospinning processes a high surface area, results in rapid evaporation of the solvent^5^.

In the current study, the effect of different production methods on the color of gelatin-chitosan film was significantly different in the experiment as shown in Table. 1 (P < 0.05). The L*, a*, b*, and ΔE* of the composite film after adding DAS were significantly different from those of films without DAS. The sensory properties and acceptance of food are affected by color. It can be concluded that the difference between the L* and ΔE* values depends on the processing conditions^21^. All DHA-enriched films’ luminance value were higher than those of other films when the luminance control value was formed by electrospinning (*P* < 0.05). Brightness is an important edible film color parameter affected by the DHA source type compound, and the form of the biologically active ingredient determines this difference. However, the possibility of using DHA source components to achieve higher L* values or brighter edible film output can be considered an advantage^22^.

Determining the optical properties of thin films and coating application capabilities on the food surface is critical, because these will influence the appearance of the coating product, which is a necessary quality factor^23^. All edible films exhibited excellent UV barrier properties in the ultraviolet range of 200–250 nm regardless of type and essential DAS incorporated (see Fig. 2), which causes oxidative deterioration of food packaging, resulting in discoloration, nutrient, loss, and odor^24^. This result was in consistent with those of previous reports on gelatin-chitosan films^25^, which demonstrated that protein-based films were believed to have high ultraviolet barrier properties, such as high content of the UV-absorbing aromatic amino acids tyrosine and tryptophan. The reason that the transmittance of GC-DTE was significantly lower than that of GC-DE was the unique combination of core and wall material in coaxial electrospinning. The transmittance of GC-DE film, in which DHA algal oil powder was directly mixed with gelatin-chitosan by electrospinning, was higher than that of GC-DTE film, in which DHA algal oil powder as a core material was combined with gelatin-chitosan,and the electrospun film without DHA algal oil powder had the lowest light transmittance. The increase in the transparency of the edible film was associated with the addition of DAS, and the color component of DAS and the destruction of the ordered membrane protein network also reduced the transparency of the edible film containing DAS^26^. The addition of DAS had an influence on the appearance and light-blocking properties of the edible films composed by different modes, and in particular, the ultraviolet barrier property was enhanced, which was beneficial to slow down the deterioration of DHA algal oil powder caused by ultraviolet radiation. In practice, the color of the packaged food and the acceptability of the color of the film should be considered in selecting different suitable edible films^27^.

Typically, edible film requires sufficient mechanical strength and ductility to withstand extrinsic stresses and keep its barrier properties and integrity during packaging applications^28^. The mechanical strength of the edible film was studied to assess the processing performance and to reveal further information about internal structures. There was a significant difference in tensile strength between GC-C and GC-DC, and the tensile strength decreased with the addition of DAS (Fig. 3A). This phenomenon may be due to the immiscibility and microphase separation of the mixture, resulting in easier chain slip under load and less physical entanglement interaction between polymer chains. Complex materials had mesoscopic microphase-separated structures, which were different from ordinary single-crystal or polycrystalline materials. Their performance depended not only on the properties of the molecule, but more importantly on the self-assembly of the molecules and the formation of microphase-separated structures from the nanometer to micrometer scale, which may be the deeper reason for the difference. Unlike other methods of adding DAS, coaxial electrospinning had the same effect as without DAS. It should be mentioned that the mechanical strength of the electrospun films was weaker than that of the casting films. The result was the same as other have authors’ reported^5^. The difference between the different methods is probably due to physical entanglement between the polymer blend of the chain interactions, resulting in the chain slides in a loading difficult^29^.

The melting temperature of the films corresponded to the dissociation of polymer chains. The endothermic peaks in the Fig. 4 mainly reflected the destruction of the hydrogen bond between gelatin molecules and the physical crosslink formed between chitosan and gelatin and DAS and the rearrangement of the gelatin’s triple helix structure. Compared to the electrospinning process, the solvent casting had a similar melting temperature, with a small gap. Higher than the melting temperature of the report of Hosseini^30^, concluded that the addition of DAS into gelatin and chitosan films, was perhaps a result of the ability of DAS to penetrate between polymer chains, thereby weakening the interaction between proteins and polysaccharides^31^. The endothermic peaks in the Fig. 4 mainly indicate destruction of the physical hydrogen bonds between the chitosan molecules and the physical crosslinking formed between the mixtures and the rearrangement of the gelatin’s triple helix structure. The increase in the thermal stability of the composite membrane and the decrease in endothermic degree are due to the bonding among gelatin and chitosan and DAS in different ways, and crosslinking is an important way of bonding^32^. Moreover, the triple helix content or crystallinity were reflected through the characteristic endothermic peaks, which were consistent with the XRD results.

X-ray diffraction analysis is a method of analyzing the internal atomic space structure of a substance by utilizing the diffraction effect of X-rays in the crystal material. However, the crystallinity of the film formed by electrospinning was significantly lower than that of the film formed by casting, and the film formed by coaxial electrospinning had the lowest crystallinity. Moreover, that of the film with DAS added was significantly lower than that of the film without DAS under the same conditions, indicating that the addition of DAS may reduce the crystallinity of the gelatin-chitosan film. The reduced peak intensity may be due to intermolecular crystal between the hydroxyl group of the DAS gelatin and NH_2_ side chain groups of the interaction, which limited the movement of molecules and thus prevented crystallization^33^.

According to Kittiphattanabawon *et al*.^34^, the amide A band usually appears in the range of 3400–3440 cm^−1^ and is related to the N–H free stretching vibration, whereas C–H stretching vibrations of aliphatic groups at 2914–2929 cm^−1^ are the amide B. The amide I of all edible films with the strong absorbance feature was mainly connected with C=O stretching vibration, which was related to coupling to CN stretching, CCN deformation, and the contribution of in-plane NH bending modes^35^. C–N stretching was in the range of 1544–1551 cm^−1^ (amide II), C–N stretching and N-H bending were a combination band, CH_2_ bending (scissors) vibration and stretching were at 1438–1453 cm^−1^, CH_2_ stretching was at 1378–1394 cm^−1^, and C–N stretching and N–H bending were at 1229–1236 cm^−1^ (amide III)^36^. The amplitude of the peaks varied with the uniform mixing of the gelatin/chitosan edible film in different ways^37^. However, the position at amide I shifted to a higher wave number for GC-C and GC-DC because of the NH group which was connected with the possible additional interactions that occurred in the chitosan, gelatin and DAS structure during casting^38^. The amide I band is acute changes in the protein secondary structure region, and the amide II band is the torsional vibration peak of the α-helix, β-sheet, corner, and random coil; amides I and II reflect the degree of cross-linking between the peptide chains: the tighter the peptide chain binding, the greater the frequency of vibration. Moreover, the amide III band reflects whether the protein maintains a complete triple helix structure.

The mechanical properties, barrier properties and thermal stability of the edible film depend to a large extent on the uniformity of macromolecules, the intermolecular forces and the solubility of the molecules, especially for the microstructure. The surface structure of the film can be observed more intuitively by scanning electron microscopy^10^. It showed that the electrospun gelatin and chitosan had good compatibility and strong intermolecular force, forming a granular network structure. GC-C and GC-DC showed only a few small pores, revealing that the gelatin chain did not aggregate in the molecule because of the presence of chitosan, so the solvent may easily leave the polymer phase without generating pores^39^. In contrast, in the electrospinning process, when the polymer is injected and accelerating toward the collector, becaues of the high surface area, rapid evaporation of the solvent occurs. It is believed that the solvent evaporation of electrospinning is based on droplets, whereas casting drying is surface based^40^.

## Conclusions

In this study, DHA powder-enhanced gelatin-chitosan edible film was prepared by casting, electrospinning and coaxial electrospinning, respectively. The electrospinning process and the coaxial electrospinning process produced a smooth surface visible to the naked eye with a uniform granular network structure in a unique film-forming manner, thereby exhibiting good water solubility and mechanical properties. In contrast, the casted film was smooth and transparent, and mechanically strong but poorly water soluble. Moreover, it was found that the addition of DHA powder affected the optical, physical, and mechanical properties of the film to varying degrees. These results indicated that these films can likely be used as activated materials for food packaging, and further research is required to define the application of these films in commercial food institutions, with potential applications value for controlled release in the biomedical field.

## Materials and Methods

### Materials and chemicals

Flounder bones were provided by Dalian Tianbao Green Foods Co., Ltd. (Dalian, Liaoning, China). Gelatin extractION from the flounder bones was performed by hydrolysis of type I collagen. The molecular weight was about 200 kDa, and the isoelectric point was around 6.7. The protein content of gelatin after removal of the heteroprotein was 32.53%, the gelatin’s pH was 7.4, and the proline and hydroxyproline concentrations were 385.89 and 232.35 nmol/mg, respectively. DHA algal oil powder was provided from Jiabiyou Biotechnology Co., Ltd. (Wuhan, Hubei, China). Chitosan was bought from Solarbio Science & Technology Co., Ltd (Beijing, China) (degree of deacetylation ≥90.0%). All other chemicals were purchased from Jinzhou Sinopharm Chemical Glass Co., Ltd. (Jinzhou, China), and all were of analytical grade.

### Preparation of gelatin from flounder bones

Gelatin was extracted from flounder bones according to the method of Ahmad *et al*.^41^ with some modifications. The following operations were performed at room temperature unless otherwise specified. The treated bones were cut into small segments (~1 cm). Bone segments were soaked in 0.1 M NaOH solution (1:30 w/v) to remove noncollagenous proteins and the solution was changed every 2 h three times. Then, the bones were defatted with 10% isopropanol solution (1:30 w/v) for 12 h with slight stirring. The treated bones were soaked in 0.5 M EDTA-2Na solution (1:30 w/v)^42^ to decalcify them and the solution was changed every 6 h five times at 4 °C. The decalcified bones were soaked in 0.1 M glacial acetic acid solution (1:10 w/v) for 4 h with slight stirring to swell the collagen in the flounder bone and returned to neutral pH (6.5~7.0) (pH meter, Hangzhou Special Paper Co., Ltd, Zhejiang, China). The swollen flounder bones were soaked in distilled water at 55 °C for 8 h with successive stirring to extract gelatin. The mixture was centrifuged (Sorvall Stratos Centrifuge, Thermo Fisher Scientific, Waltham, MA, USA) at 8000 g for 20 min and the sediment was discarded. The gelatin supernatant was freeze-dried using a vacuum freeze dryer (FreeZone 2.5 L, Labconco, Palo Alto, CA, USA).

### Preparation of film-forming solutions

A flounder bone gelatin solution was prepared based on the method of Nilsuwan *et al*.^43^ with a slight modification. Gelatin powder was mixed with 80% glacial acetic acid to obtain a concentration of 8% (w/v). The mixture was heated at 55 °C in a water bath (HH-6 Digital Display Thermostatic Bath, Changzhou Guohua Electric Appliance Co., Ltd, Jiangsu, China) for 20 min with continuous stirring to completely solubilize the gelatin, and cooled to 45 °C. Glycerol (0.4 g/g gelatin) was added to the gelatin as a plasticizer. Simultaneously, chitosan solution was made to a 2% (w/v) concentration in 80% glacial acetic acid. The DHA powder was mixed with 80% glacial acetic acid to obtain a concentration of 10% (w/v) (DAS). The gelatin solution and chitosan solution were mixed at a ratio of 1:1 (w/w) (GCS) and then successively stirred until the solution complete dissolution.

### Casting process

DAS was added to GCS at a concentration of 5% (v/v). Then, 30 mL solutions were poured on aluminum foil and desiccated at 30 °C in an oven (DHG-9055A blast drying oven, Shanghai Yiheng Scientific Instrument Co., Ltd, Shanghai, China) for 24 h. The thickness of the film was controlled in the range of 0.15 ± 0.05 mm. The films (GC-C and GC-DC; D in the abbreviation represents docosahexaenoic acid and C represents the casting process) were held in a dryer (MZ250 Vacuum Desiccators, Shanghai Experi-mental Instrument Company, Shanghai, China) at a room temperature of 25 °C.

### Electrospinning process

An electrospinning device (Beijing Ucalery Technology Development Co., Ltd, Beijing, China) was used in the process. The solution was the same as the solution for the casting process. A syringe with solution was equipped with a 23-G steel needle and the solution was sprayed at a 0.1 mm/min velocity of flow. The positive and negative voltages were 22 and 2 kV, respectively; the distance from the sprinkler to the receiving device was 11 cm; and the receiving speed of the roller receiver and translation speed of the spraying device were 20 r/min and 200 mm/min respectively. The spin-coated film was collected on a flat piece of aluminum foil. The electrospinning condition was held at 30 °C, and the thickness of the film (GC-E and GC-DE, E in the abbreviation represents the electrospinning process.) was controlled in the range of 0.15 ± 0.05 mm.

### Coaxial electrospinning process

Different from electrospinning, 2 syringes for coaxial electrospinning were used. Syringe A was loaded with core material, and syringe B was loaded with wall material. The core material was DAS and the speed of syringe A was 0.05 mm/min; the wall material was GCS and the speed of syringe B was 0.1 mm/min. Other conditions were the same as those of the electrospinning process. The films (GC-DTE; TE in the abbreviation represents the coaxial electrospinning process.) were kept in a vacuum dryer at a room temperature of 25 °C.

### Water solubility

The films were cut into 10 × 40 mm pieces and dried in a desiccator to a constant weight, and the film quality *W*_*i*_ was weighed. The sample was immersed in 30 mL water, kept at room temperature for 24 h, and dried to a constant weight in a 60 °C blast oven, and the film quality *W*_*f*_ was weighed.

Water solubility was calculated as follows^44^:

$${\rm{FS}} \% =({W}_{i}-{W}_{f})/{W}_{i}\times 100 \% $$

### Color measurement

The color (L*, a*, b*, and ΔE*) of the edible films was measured by a chromameter (CR-400, Konica Minolta, Tokyo, Japan) and calibrated with a white standard plate (L* = 93.25, a* = −5.21, b* = 7.68). Five positions were chosen randomly on each sample’s surface and averaged. The total color difference (ΔE*) was calculated by the following equation^45^:

$${\rm{\Delta }}{E}^{\ast }={[{({\rm{\Delta }}{L}^{\ast })}^{2}+{({\rm{\Delta }}{a}^{\ast })}^{2}+{({\rm{\Delta }}{b}^{\ast })}^{2}]}^{0.5}$$

### Transparency and light transmission

A UV-visible spectrophotometer (Shimadzu UV-2550, Japan) was used to test the barrier properties of the edible film in the wavelength range of 200–800 nm. The film was cut into 10 × 40 mm samples and attached to the side of a cuvette and a blank cuvette was used for reference. The absorbance was measured at a wavelength of 600 nm to determine the transparency of the film^46^. Each sample was assayed for 3 times. The transparency was calculated as follows:

$${\rm{Transparency}}=-\,\mathrm{log}\,{\rm{T}}\,600/{\rm{x}}$$

In the formula: Transmittance of the edible film at T 600: 600 nm; x: Thickness of film/mm.

### Mechanical strength

The mechanical strength of the films was tested using a TA-XT plus a texture analyzer with a 5-N-capacity load cell (Stable Micro Systems Ltd, Godalming, UK). The edible film was cut into rectangular strips of 50 × 20 mm and used for measuring the maximum tensile force and tensile strength at break. The samples were tested in 2 cycles at a test speed of 3.0 mm/s, pre-test speed of 1.0 mm/s, and post-test speed of 10.0 mm/s. The initial test pitch was 30 mm. Each sample was repeated 3 times, and the reading data were averaged prior to the statistical analysis.

The tensile strength was calculated as follows:

$${\rm{TS}}({\rm{MPa}})={\rm{F}}/{\rm{S}}$$

In the formula, F was the maximum tensile force when the edible film broke, and S was the cross-sectional area of the edible film.

Elongation at break was calculated as follows:

$${\rm{E}} \% =({\rm{L}}^{\prime} -{\rm{L}}^{\prime\prime} )/{\rm{L}}^{\prime\prime} \times 100 \% $$

In the formula, L′ was the length of the edible film at the time of the break, and L″ was the initial length of the edible film.

### Differential scanning calorimetry (DSC)

The denaturation temperatures of the edible film were measured with a differential scanning calorimeter (Q2000, TA Instruments, New Castle, USA). Seven mg of the sample was weighed into a DSC aluminum pan and sealed with a lid. The temperature was held at 20 °C for 1 min, with a heating rate of 5 °C/min heated from 20–90 °C and using an empty aluminum pan as a reference. The denaturation temperature was the temperature at the maximum point of the peak estimated using the TA Universal Analysis software (Q2000, TA Instruments, New Castle, USA), and the converted enthalpy was the area under the peak.

### X-ray diffraction

The crystal structures of the edible films were investigated using a Rigaku Ultima IV X-ray diffraction instrument (Rigaku Corporation, Tokyo, Japan). The scan speed was 10°/min using Cu Kɑ radiation with a test range from 5–50°(2θ). The X-ray generator had a tube current of 40 mA and an operating voltage of 40 kV. The experiments were performed in triplicate.

### Fourier transform infrared spectroscopy (FTIR)

The composition of the secondary structure of edible films was analyzed by a Scimitar 2000 Near FTIR Spectrometer (Scimitar 2000, Madison, WI, USA). The wave number ranged from 4000 to 400 cm^−1^, and the resolution was 2 cm^−1^ with 32 scans. The sample (1 mg) and the dried KBr (100 mg) (Muyonga, Cole, & Duodu, 2004b) were respectively placed in an agate mortar and ground to a uniform powder, loaded into a sample, and placed in a sample chamber for scanning. All FTIR experiments were performed in triplicate and used the software provided with the instrument to obtain the average spectrum.

### Scanning electron microscope (SEM)

The microstructure of the edible film was observed by a scanning electron microscope (S-4800, Hitachi, Tokyo, Japan). A suitable amount of the regular shape of the edible film sample was subjected to gold plating by ion sputtering (E-1045 ion sputter, Hitachi, Tokyo, Japan) to make the sample conductive and the surface structure was observed by magnifying by 1.0 k at a voltage of 3.0 kV.

### Statistical analysis

Univariate analysis of variance (ANOVA) analysis of all quantitative data (P < 0.05), followed by the Duncan multiple range test, expressed as mean ± standard deviation (SD) (SPSS 22.0, SPSS Inc., Chicago, IL), USA). All experiments were performed in triplicate, using three lots of different films. All figures drawn using OriginPro 9.0 (OriginLab Co., Northampton, MA, USA). The datasets generated and/or analyzed during the current study are available from the corresponding author on reasonable request.
